# The Role of microRNAs in the Regulation of Apoptosis in Lung Cancer and Its Application in Cancer Treatment

**DOI:** 10.1155/2014/318030

**Published:** 2014-06-05

**Authors:** Norahayu Othman, Noor Hasima Nagoor

**Affiliations:** ^1^Division of Genetics and Molecular Biology, Institute of Biological Sciences, Faculty of Science, University of Malaya, 50603 Kuala Lumpur, Malaysia; ^2^Centre for Research in Biotechnology for Agriculture (CEBAR), University of Malaya, 50603 Kuala Lumpur, Malaysia

## Abstract

Lung cancer remains to be one of the most common and serious types of cancer worldwide. While treatment is available, the survival rate of this cancer is still critically low due to late stage diagnosis and high frequency of drug resistance, thus highlighting the pressing need for a greater understanding of the molecular mechanisms involved in lung carcinogenesis. Studies in the past years have evidenced that microRNAs (miRNAs) are critical players in the regulation of various biological functions, including apoptosis, which is a process frequently evaded in cancer progression. Recently, miRNAs were demonstrated to possess proapoptotic or antiapoptotic abilities through the targeting of oncogenes or tumor suppressor genes. This review examines the involvement of miRNAs in the apoptotic process of lung cancer and will also touch on the promising evidence supporting the role of miRNAs in regulating sensitivity to anticancer treatment.

## 1. Introduction

Lung cancer remains a major health problem worldwide. In 2012 lung cancer was the most commonly diagnosed cancer worldwide making up 13.0% of the total incidence of cancer. It was also the most common cause of death from cancer worldwide, accounting for nearly one in five cancer deaths (19.4% of the total) [1]. Lung cancer is clinically divided into two main groups based upon the size and appearance of malignant cells: small cell lung cancer (SCLC) (16.8%) and non-small cell lung cancer (NSCLC) (80.4%) [2]. The most effective option for treatment of lung cancer is surgical resection, when feasible [3]. However, majority of patients are diagnosed at an advanced or metastatic stage of disease in which case chemotherapy and/or concurrent administration of chemotherapy and radiation is the most beneficial form of treatment [4]. Nevertheless, even with treatment, the 5-year survival rate in patients is only 16.6% [5], with poor survival rates mainly being attributed to late stage diagnosis and high frequency of drug resistance. Obtaining a better understanding regarding the molecular mechanisms involved in lung carcinogenesis is of utmost importance in the aim to identify the diagnostic and prognostic markers for early detection and targeted treatment of lung cancer.

Apoptosis plays an important role during development and in the maintenance of multicellular organisms through the removal of damaged, aged, or autoimmune cells [6]. The apoptotic process can be divided into the extrinsic and intrinsic pathway. Each pathway will ultimately result in the activation of cell death proteases, which in turn initiates a cascade of proteolysis involving effector caspases that carries out the completion of the apoptotic process [7]. In contrast to normal cells, cancer cells have the ability to evade apoptosis to promote cell survival under the conditions of environmental stress. There are a number of mechanisms by which cancer cells are able to suppress apoptosis. For example, the tumor suppressor gene* p53* is a widely mutated gene in human tumorigenesis [8].* p53* mutation will inhibit the activation of DNA repair proteins leading to a decrease in the initiation of apoptosis [7], allowing for cells to divide and grow uncontrollably, forming malignant tumors. Furthermore, cancer cells are able to disrupt the balance between pro- (*BCL-2*,* BCL-XL*) and antiapoptotic factors (*BAX*,* BIM*, and* PUMA*) [9]. Increased expression of proapoptotic Bcl-2 protein contributes not only to the development of cancer but also to resistance against a wide variety of anticancer agents, such as cisplatin (DDP) and paclitaxel [10–12].

**Figure 1:**
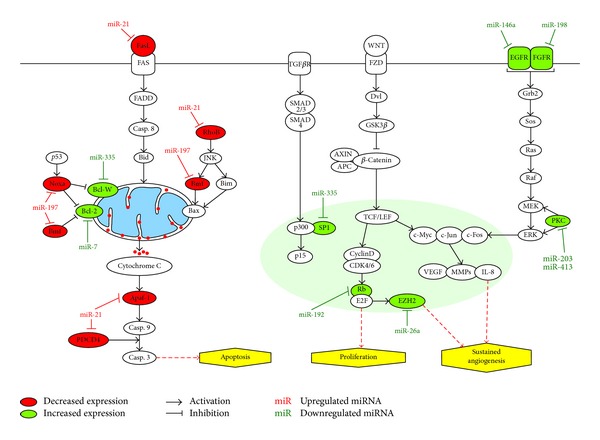
Scheme depicting up- and downregulated miRNAs and the roles they play in various biological pathways including apoptosis, proliferation, and angiogenesis.

MicroRNAs (miRNAs) are a subset of noncoding RNAs of about 20 to 25 nucleotides long which posttranscriptionally regulate gene expression via inhibition of mRNA translation, by binding to specific target sites in their 3′-untranslated region (3′UTR), or inducing degradation of target mRNA through cleavage [13]. An individual miRNA is able to modulate the expression of multiple genes; correspondingly, a single target can be modulated by many miRNAs [14]. MiRNAs were reported to be involved in a vast range of biological processes, including apoptosis (see Figure 1) [15–22]. As miRNAs play a key role in an assortment of biological processes, an altered miRNA expression is likely to contribute to human diseases including cancer [23]. Previous studies have shown that compared to normal tissues, malignant tumors and tumor cell lines were found to have widespread deregulated miRNA expression [24–28]. MiRNAs are critical apoptosis regulators in tumorigenesis and cancer cells are able to manipulate miRNAs to regulate cell survival in oncogenesis. Many studies carried out in the past several years are aimed at elucidating the specific miRNAs associated with apoptosis in cancer and their related target genes. In this review we will examine the recent progress of research on miRNA-mediated regulation of apoptosis in lung cancer and its future therapeutic applications.

## 2. Antiapoptotic miRNAs

Evasion of apoptosis is a significant hallmark of tumor progression, and one mechanism by which miRNAs influence development of cancer is through the regulation of the apoptotic process as shown in various studies [29–32]. miRNA expression can be either upregulated or downregulated and evidence has shown that dysregulated miRNAs can behave as oncogenes or tumor suppressor genes in lung cancers [18, 28, 33]. Amplification of miRNAs can lead to the downregulation of tumor suppressors or other genes that are involved in apoptosis [34].

*miR-197*. For example, the expression of miR-197 is increased in cancer tissues in comparison to normal specimens. Fiori et al. (2014) demonstrated that knockdown of miR-197 in NIH-H460 and A549 cells promoted induction of apoptosis, evident by the observation of caspases 3–7 activation and increased apoptotic population by Annexin staining. Furthermore, the direct interaction of miR-197 with the 3′UTR of* BMF* and* NOXA *was demonstrated by the luciferase reporter assay [35]. When activated by intra- or extracellular stimuli, proapoptotic Bmf binds to and neutralizes antiapoptotic Bcl-2 family members on the mitochondrial membrane, thus allowing proapoptotic proteins Bak and Bax to dimerize and promote the release of cytochrome c, ultimately leading to cell death [36]. Noxa is a BH-3 only proapoptotic protein transcriptionally activated by* p53*. Collectively, miR-197 is able to act upon different levels of the* p53* pathway to counteract the induction of apoptosis, thus allowing cells to proliferate uncontrollably [35].

*miR-21*. miR-21 is found to be frequently upregulated in a number of cancers; however its potential role in tumorigenesis* in vivo* is not fully explored. Using transgenic mice with loss-of-function and gain-of-function miR-21 alleles, Hatley and colleagues elucidated the role of miR-21 in NSCLC pathogenesis* in vivo *[37]. It was determined that miR-21 regulates tumor proliferation and survival, which are two integral components of NSCLC pathogenesis, by targeting negative regulators of the RAS pathway as well as by targeting proapoptotic genes [37]. In regards to the apoptotic pathway, overexpression of miR-21* in vivo* leads to decreased protein levels of Apaf-1, an important component of the intrinsic mitochondrial apoptotic pathway, as well as decreased expression of FasL, a key initiator of the extrinsic apoptotic pathway. Furthermore,* RHOB*, with a tumor suppressor role, is a target of miR-21 and its dysregulation leads to an increase in cell growth and inhibition of apoptosis [38]. Together these results suggest that relieving miR-21 downregulation of these proapoptotic and tumor suppressor genes could provide a means to enhance the effect of current chemotherapy.

*miR-212*. Acetylcholinesterase (AChE), a component of the cholinergic system, has the ability to influence apoptotic sensitivity both* in vitro* and* in vivo *[39–41]. In NSCLC tissues AChE levels are low and are associated with tumor aggressiveness, increase risk of postoperative recurrence, and low survival rate [42]. Lu et al. (2013) determined that* AChE* expression in NSCLC was posttranscriptionally modulated by miR-212 binding to its 3′UTR. Interestingly, alterations in neither AChE nor miR-212 expression significantly affected cell survival; however it was observed that during DDP-induced apoptosis miR-212 levels were reduced with a concurrent increase in AChE protein levels. This suggests that miR-212 plays a role in DDP resistance by directly inhibiting AChE and preventing apoptosis. Therefore, interference against miR-212 may potentially be a means to improve the pharmacotoxicological profile of DDP in NSCLC [43].

*miR-17-5p and miR-20a.* The miR-17-92 cluster, which is composed of seven miRNAs and resides in intron 3 of the* C13orf25* gene at 13q31.3, is frequently overexpressed in lung cancers [44]. Matsubara et al. (2007) demonstrated that inhibition of two components of the miR-17-92 cluster, miR-17-5p, and miR-20a, with antisense oligonucleotides can induce apoptosis selectively in lung cancer cells that overexpress miR-17-92 [45]. Previously, miR-17-5p and miR-20a have been shown to directly target* E2F1* [46]; thus inhibition of these miRNAs may cause the induction of apoptosis in part through the induction of* E2F1* and subsequent cell cycle progression into* S* phase [47]. However additional studies would have to be carried out to determine the actual targets for the miR-17-92 cluster to gain a better understanding of the development of this cancer.

## 3. Proapoptotic miRNAs

MiRNAs that are downregulated are considered tumor suppressor genes. Tumor suppressor miRNAs usually prevent tumor development by negatively regulating oncogenes and/or genes that control cell differentiation or apoptosis [48]. MiRNAs that act as tumor suppressors can be downregulated as a result of deletions, epigenetic silencing, or loss of expression of transcription factors (see Table 2) [49].

### 3.1. B-Cell Lymphocyte 2 (*BCL-2*) Family Related miRNAs

Members of the evolutionarily conserved* BCL-2* family are thought to be the central regulators of apoptosis. The expression level of* BCL-2* differs for different cell types; however high levels and aberrant patterns of* BCL-2* expression were reported in a wide variety of human cancers, including lung cancer [50]. Elevation of Bcl-2 protein expression contributes not only to the development of cancer but also to resistance against a wide variety of anticancer agents [10–12].

*miR-7*. Xiong et al. showed that miR-7 was downregulated in NSCLC cells and* BCL-2* was identified as a direct target [51]. Transfection of miR-7 in A549 cells led to a significant reduction in endogenous* BCL-2* mRNA and protein levels and correspondingly led to increase in the activities of caspase-3 and caspase-7 in cells with apoptotic nuclei [51]. These results thus provide evidence that* BCL-2* may be involved in miR-7 mediated apoptosis induction in A549 cells.

*miR-335*.* BCL-W*, another antiapoptotic member of the* BCL-2* family, was found to be a direct target of miR-335 [52]. miR-335 was downregulated in A549 and NCI-H1299 cells, and upregulation of this miRNA via transfection of miR-335 mimics led to a suppression of cell invasiveness and promotion of apoptosis. Furthermore Dyanan and Tjian (1983) discovered that miR-335 directly targeted* SP1* gene, a member of the family of Sp/Kruppel-like transfection factors [53], which can enhance the activity of promoters of numerous genes involved in cell proliferation, apoptosis, differentiation, cell cycle, progression, and oncogenesis thus regulating these genes' expression [54].

*miR-608*. Studies in our lab identified a* BCL-XL*-induced miRNA, miR-608, involved in the regulation of cell death in A549 and SK-LU-1 cells [55].* BCL-XL*, a major prototype of the antiapoptotic* BCL-2* gene was found to be overexpressed in NSCLCs [56]. Silencing of* BCL-XL* in A549 and SK-LU-1 led to the significant dysregulation of a number of miRNAs, as determined through miRNA microarray, with miR-608 being the most upregulated miRNA. Upregulation of miR-608 in A549 and SK-LU-1 via miR-608 mimics led to an increase in apoptotic population, as determined by Annexin-V FITC apoptotic assay, in comparison to NP-69 cells (normal human nasopharyngeal epithelial cell line) (see Table 1) [55]. Bioinformatics analysis determined that miR-608 may be associated with various signaling pathways, primarily the phosphatidylinositol 3-kinase/protein kinase B (PI3K/AKT), wingless-type MMTV integration site family (WNT), transforming growth factor (TGF-*β*), mitogen activated protein kinase (MAPK), and the intrinsic pathway. However the true targets of miR-608 and its direct effects on the apoptotic process is yet to be determined.

### 3.2. Protein Kinase C (PKC) Family Related miRNAs 

PKC is a serine/threonine kinase that is involved in various signal transduction pathways including those related to cellular proliferation, differentiation, and apoptosis [57–59]. PKC plays a role in lung cancer and levels of PKC proteins were found to be increased in various cell lines (A549, NCI-H1355, NCI-H1703, NCI-H157, and NCI-H1155) in comparison to primary normal human bronchial epithelial cells (NHBE) [60].

*miR-203*. To determine the role that miR-203 can play in the influence of cellular function, putative target prediction was carried out and PKC-**α** was determined to be a target [61]. Luciferase reporter assay further revealed miRNA-203 direct binding of the 3′UTR of* PKC-*α** mRNA transcript. miR-203 negatively regulated proliferation and migration through the repression of* PKC-*α**, and miR-203 was also able to modulate cell apoptosis. However, siRNA silencing of* PKC-*α** resulted in a less significant apoptotic phenotype in comparison to that observed by miR-203 overexpression, thus suggesting that miR-203 may modulate multiple apoptotic genes that work together to regulate cell apoptosis [61]. Further studies must therefore be carried out to determine the additional apoptosis related targets of miR-203.

*miR-143*. miR-143 expression was reported to be downregulated in cancer tissues and inhibition of miR-143 promotes cell proliferation but hinders cell apoptosis. To determine the role that miR-143 plays in the apoptotic process, Akita (2002) investigated the possible targets of miR-143 and found that PKC-**ε**, a crucial enzyme in various cellular signaling pathways [62], was a putative target. Using the luciferase reporter assay it was determined that miR-143 specifically targets* PKC-*ε**, and overexpression of miR-143 increases the cell apoptosis in A549 cells [63]. PKC-*ε* was suggested to play a role in regulating the antiapoptotic signaling pathway through the upregulation of Bcl-2 with a concurrent suppression of proapoptotic Bid [64–66]. Furthermore, PKC-*ε* is able to activate Akt to apply its prosurvival effects [67, 68]. Therefore, the targeting of* PKC-*ε** could potentially be a valuable therapeutic strategy for lung cancer.

### 3.3. Other miRNAs

*miR-198*. miR-198 is downregulated in NSCLC cell lines and overexpression of this miRNA inhibits cell viability and enhances apoptosis in A549 cells. Overexpression of miR-198 induces the expression of poly(ADP-ribose) polymerase (PARP) and of cleaved caspase-3. miR-198 was also able to inhibit growth of tumor grafts in nude mouse.* FGFR1*, a lung cancer oncogene, which is a membrane-bound receptor tyrosine kinase that regulates proliferation via the MAPK and PI3K pathway, much like EGFR, was found to be a direct target of miR-198 [69].

*miR-146a*. Expression of miR-146a is low in malignant tissues in comparison to corresponding adjacent normal lung tissues. Functionally, miR-146a suppresses cell growth, inhibits cell migration and increases cellular apoptosis [70]. Upregulation of miR-146a expression via miR-146a mimic transfection resulted in the downregulation of EGFR as well as phosphorylated EGFR, both at the mRNA and at protein levels. Furthermore, downstream pathways (ERK-1/2, AKT, and STAT) were also downregulated in response to miR-146a mimic transfection, albeit with a weaker effect as that seen by cells transfected with* EGFR* specific siRNA. miR-146a mimic also led to the decrease of phosphorylation of the NF-*κ*B inhibitor I*κ*B*α*, but not total I*κ*B*α*. Levels of phospho-NF*κ*B, total NF-*κ*B, and the total immune-modulating kinase, IRAK-1, were also found to be decreased following miR-146a mimic transfection, suggesting that miR-146a regulates NF-*κ*B and IRAK-1 signaling [70].

*miR-26a*. miR-26a expression is downregulated in lung cancer tissues relative to normal tissues. Transfection of miR-26a into A549 cells was able to decrease cell proliferation, block the G1/S phase transition of cell cycle, and induce apoptosis [71]. The chromatin regulator enzyme EZH2, which regulates survival and metastasis of cancer cells [72], was found to be a direct target of miR-26a. Downregulation of* EZH2* expression, caused by overexpression of miR-26a will transactivate downstream tumor suppressor genes* DAB21P* and* RUNX3*. DAB21P is a potent growth inhibitor that induces G0/G1 phase cell cycle arrest and could lead to apoptosis [73], while RUNX3 leads to cell cycle arrest, apoptosis, and significant decrease of tumor growth and abrogation of metastasis [74].

*miR-451*. Poor tumor differentiation, advance pathological state, lymph node metastasis, and poor prognosis are associated with downregulation of miR-451, which occurs in lung cancer [75]. To observe the functions of miR-451, Wang et al. (2011) upregulated miR-451 expression via mimics and observed suppressed* in vitro* proliferation, chromatin condensation and nuclear fragmentation upon 4′,6-diamidino-2-phenylindole (DAPI) staining, and significant caspase-3 activity. These results suggested that ectopic expression of miR-451 was able to induce an increase in apoptosis in a caspase-3 dependent manner. In addition, the* RAB14 *gene was identified as a direct target of miR-451. Inhibition of* RAB14* led to a decrease in phosphorylation of Akt, which subsequently decreased levels of Bcl-2 protein expression and increased proapoptotic Bax or Bad protein expression. As the expression levels of RAB14 protein were inversely correlated with the expression levels of miR-451 in NSCLC tissues it was concluded that downregulation of RAB14 may be the mechanism by which miR-451 carries out its tumor suppressor functions [75].

*miR-192*. miR-192 was found to be downregulated in A549, NCI-H460, and 95D cell lines [76]. Cell viability was greatly decreased following miR-192 upregulation, while levels of apoptosis were elevated with induced expression of PARP protein and cleaved caspase-7, thus suggesting that miR-192 induces apoptosis through the caspase pathway. Using bioinformatics analysis,* RB1* gene was determined to be a putative target of miR-192 and luciferase reporter assays confirmed direct binding of miR-192 to the 3′-UTR of this gene [76]. Since RB1 plays a vital role in regulating cell apoptosis, its downregulation was shown to induce *γ*-H2AX foci formation, a marker of DNA damage, and to promote apoptosis in A549 cells [77].

## 4. miRNA and Response to Cancer Therapy

**Figure 2:**
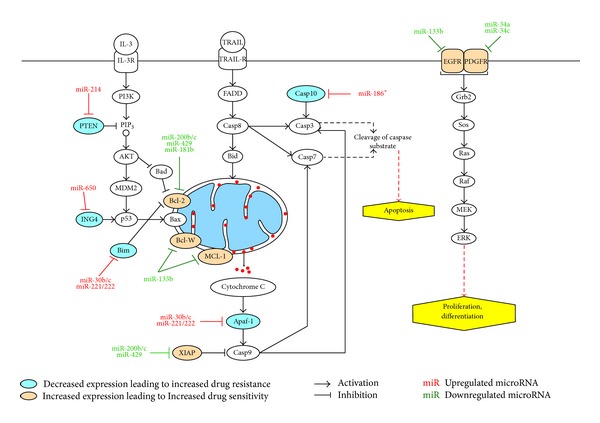
Scheme depicting the roles miRNAs play in sensitivity and resistance to common cancer treatments.

Many cancer therapies available today aim to induce tumor-selective cell death; however resistance to chemotherapeutics is a significant obstacle to the long-term treatment and survival of NSCLC patients [78]. Presently, there are various chemotherapeutics that are being utilized in the treatment of lung cancer, including FDA approved drugs (DDP, paclitaxel, docetaxel, gemcitabine, and EGFR-TKIs), natural compounds (curcumin), and small organic compounds (PRIMA-1) (see Table 3). The association of miRNAs as regulators of malignancy and apoptosis has been widely reported; thus it is reasonable to assume that miRNAs play significant roles in sensitivity/resistance to common cancer treatments (see Figure 2) [79]. Indeed, recent studies have demonstrated miRNAs as potential agents involved in the sensitivity of lung cancer cells to cytotoxic therapy.

### 4.1. Cisplatin- (DDP-) Related miRNAs

DDP is a platinum-coordinated complex that is the most widely used chemotherapy for human NSCLC in the past two decades [80–82]. However, multiple administration of DDP results in the development of drug resistance leading to failure of treatment, as demonstrated by tumor growth or tumor relapse [78, 83]. Therefore, to overcome the treatment plateau of DDP on NSCLC, the biological mechanisms by which DDP action is enforced must be further elucidated. As miRNAs act as critical regulators in the development of drug resistance, it would be interesting to research the mechanism through which oncogenic miRNAs modulates DDP-induced apoptosis in NSCLC.

*miR-451.* miR-451 was downregulated in NSCLC tissues in comparison to normal lung tissues, and upregulation of miR-451 enhances DDP chemosensitivity in A549 cells by inhibiting cell growth and inducing apoptosis enhancement [84]. Bian et al. (2011) demonstrated in their study that upregulation of miR-451 enhanced caspase-3-dependent apoptosis through the inactivation of the Akt signaling pathway, which in turn decreased Bcl-2 while increasing expression of Bax protein levels. Furthermore, results of Annexin V-FITC apoptosis assay indicated that in miR-451 transfected A549 cells (A549/miR-451) a higher percentage of apoptosis was observed in comparison to mock A549 cells. Caspase-3 activity in A549/miR-451 treated with DDP was significantly increased against the control, thus suggesting that miR-451 upregulation increases chemosensitivity of A549 cells by enhancing DDP-induced apoptosis. Together these results suggest a possible strategy for treatment of human NSCLC through the combined application of DDP treatment with miR-451 upregulation [84].

*miR-31.* On the other hand, miR-31 is upregulated in NSCLC cell lines and was demonstrated to induce DDP resistance. To demonstrate this, Glavinas et al. (2004) transfected miR-31 mimics into DDP-sensitive SPC-A-1 cells which led to a marked increase in the resistance of SPC-A-1 cells, while transfection of miR-31 inhibitors increased sensitivity of resistant NCI-H1299 to DDP treatment. To elucidate the mechanism by which DDP resistance is induced by miR-31, bioinformatics analysis was carried out and* ABCB9*, a membrane transporter involved in drug uptake [85], was predicted to be a target gene. The luciferase reporter assay then confirmed direct miR-31 regulation of* ABCB9* by binding to its 3′UTR [86]. Overexpression/knockdown studies indicated a significant decrease in the percentage of DDP-induced apoptotic cells when miR-31 was increased via mimics and a marked increase in DDP-induced apoptotic cells when miR-31 inhibitors were introduced, thus suggesting that miR-31 exerts an antiapoptotic effect in DDP-induced apoptosis through the inhibition of* ABCB9*.

### 4.2. Paclitaxel-Related miRNAs

Paclitaxel was the first identified member of taxanes in the list of FDA-approved anticancer drugs. This compound has been shown to have significant single-agent activity against various solid tumors [87, 88] including NSCLC [89]. However, combination of this compound with DDP or carboplatin showed superior response and improved survival rates [90].

*miR-133a/b and miR-361-3p*. High-throughput screening (HTS) approach was performed by Du and colleagues in 2013 to identify miRNAs that modulate lung cancer cell survival and response to paclitaxel treatment [91]. Using three NSCLC cell lines that have distinct genetic backgrounds (NCI-H1155, NCI-H1993, and NCI-H358), inhibition of two miRNAs (miR-133a/b and miR-361-3p) was found to potently decrease cell viability, although cytotoxicity of the two miRNAs vary greatly, which may be due to different endogenous expression levels of the miRNAs in each cell line. Interestingly, the inhibitors of miR-133a/b and miR-361-3p were found to reduce cell survival through different mechanisms. miR-133a/b inhibitor was able to dramatically increase apoptotic events as seen by increased percentage of cells undergoing apoptosis and increased levels of activated caspase-3. However miR-361-3p only showed a modest effect on caspase-3 activation thus suggesting that additional mechanisms are involved in the cytotoxicity of this miRNA. The effect of miRNA inhibitors on cell cycle distribution was then evaluated and results indicated that S phase arrest contributes to cytotoxicity induced by miR-133a/b and miR-361-3p inhibitors. Together these results suggest that miR-133a/b and miR-361-3p may function as oncogenes in cancer cells by regulating tumor suppressor genes.

*miR-101*. Increasing evidence has revealed that EZH2 has oncogenic properties, as an increased expression of EZH2 augments proliferation and invasion of cancer cells [92–94], while depletion leads to a decline in cell proliferation, increased apoptosis, and inhibition of metastatic tumor growth* in vivo *[95, 96]. Overexpression of EZH2 has been associated with tumor progression and cancer aggressiveness in NSCLC [97]. In a study by Zhang and colleagues (2011), it was discovered that a decreased expression of miR-101 was associated with EZH2 overexpression in NSCLC tissues [98]. Luciferase reporter assay revealed that miR-101 regulates EZH2 expression through the binding of its 3′UTR mRNA. Overexpression of miR-101 led to a decrease in EZH2 protein levels with subsequent decrease in the proliferation and invasive ability of NSCLC cells. Furthermore, overexpression of miR-101 led to a sensitization of NSCLC cells to paclitaxel.

### 4.3. Docetaxel-Related miRNAs

Docetaxel, a semisynthetic analog of paclitaxel, is one of the first-line chemotherapy regimens for advanced NSCLC, with genotoxic effects caused by microtubule stabilizing, apoptotic induction through microtubule bundling, and Bcl-2 blocking [99, 100].

*miR-100*. In a miRNA microarray profiling carried out by Rui and colleagues in 2010, miR-100 was significantly downregulated in docetaxel-resistant SPC-A1/DTX cells relative to SPC-A1 parental cells [101]. To elucidate the role that miR-100 plays in the formation of docetaxel resistance, the authors' transfected miR-100 mimics SPC-A1/DTX cells [102]. Results suggested that restoration of miR-100 expression chemosensitizes cells to docetaxel* in vitro*, complemented with a suppression of cell proliferation, enhancement of apoptosis, and cell cycle arrest in the G2/M phase of cell cycle. Ectopic miR-100 expression was also able to downregulate* in vivo *cell proliferating ability. Moreover,* PLK1* gene was identified to be a direct target of miR-100.* PLK1* plays a role in promotion of cell proliferation and overexpression of this gene has been observed in various human cancers [103] including NSCLC [104]. Knockdown of Plk1 protein expression by miR-100 led to a significant suppression of cell proliferation of SPC-A1/DTX, dramatic increase of early apoptosis rate, G2/M arresting population, and an increase in the response of SPC-A1/DTX cells to docetaxel both* in vitro* and* in vivo*. miR-100 was therefore concluded to function as a chemosensitizer restorer to docetaxel by targeting* PLK1* and inducing the suppression of cell proliferation, enhancement of apoptosis, and mitotic arrest.

*miR-650*. High expression of miR-650 can be found in lung cancer tissues, and its dysregulation is correlated with advance clinical stage as a poor prognostic factor for these patients [105]. Furthermore, Huang et al. (2013) determined that the expression of miR-650 is negatively correlated with patients' response to docetaxel. Using two docetaxel-resistant cell lines (SPC-A1/DTX and H1299/DTX), the authors demonstrated that downregulation of miR-650 was able to reverse the resistance.* ING4*, a novel tumor suppressor gene, was then identified as the functional target of miR-650 and results from flow cytometry and Hoechst staining assays indicated that miR-650 inhibitor was able to induce an increase in caspase-3-dependent apoptosis. Cells transfected miR-650 inhibitors exhibited decreased expression of Bcl-2 protein, with an increased expression of Bax protein, led to the progression of apoptosis. The findings of this study confirmed that miR-650 was able to confer docetaxel chemoresistance through the regulation of Bcl-2/Bax expression by targeting of* ING4* [105].

### 4.4. Gemcitabine-Related miRNA

Gemcitabine, a pyrimidine nucleoside antimetabolite, has been shown to be an effective agent most particularly when administered in combination regimes [106]. Due to its theoretical ability of interfering with the inhibition of repair of platinum-induced DNA damage, gemcitabine is the perfect partner for platinum compounds. Gemcitabine in combination with DDP represents a common first-line treatment for patients with advanced NSCLC, especially in Europe [80, 107–110].

*miR-133b*. miR-133b is greatly reduced in cancer tissue in comparison to adjacent normal lung tissue [111]. Prediction programs identified two common predicted targets of miR-133b, the antiapoptotic* MCL-1* and* BCL-W*, both of which are members of the antiapoptotic* BCL-2* family [112] and have previously been reported to be increased in both solid and hematological malignancies including lung cancer [113, 114]. Transfection of miR-133b using pre-miR-133b resulted in a decrease in Bcl-W and Mcl-1 protein expression with a moderate increase of apoptosis. However combination treatment of miR-133b overexpression with 24 hours treatment of gemcitabine resulted in a greater degree of cleaved PARP expression as well as apoptosis. This concludes that miR-133b is able to target prosurvival molecules and induce apoptosis in the setting of chemotherapeutic agents [111].

### 4.5. Epidermal Growth Factor Receptor Tyrosine Kinase Inhibitors- (EGFR-TKIs-) Related miRNAs

EGFR is a plasma membrane glycoprotein that belongs to a family of four different tyrosine kinase receptors (EGFR (ErbB1), HER2/neu (ErbB2), HER3 (ErbB3), and HER4 (ErbB4)) [115]. Dimerization of EGFR may result in cancer cell proliferation, inhibition of apoptosis, invasion, metastasis, and tumor induced neovascularization [116]. Mutations and subsequent overexpression of EGFR can be found in all histologic subtypes of NSCLC [117]. Deletion in exon 19, which removes the conserved sequence LREA, and a single point mutation in exon 21, which leads to the substitution of arginine for leucine at position 858 (L858R), are the most clinically relevant and extensively studied drug-sensitive mutations [118]. Studies have shown that these mutations preferentially bind to first generation EGFR-TKIs, gefitinib and erlotinib [119, 120]. First generation EGFR-TKIs function by selectively targeting the receptor via a competitive, reversible binding at the tyrosine kinase domain, thus leading to the inhibition of ATP binding and subsequent signal transduction and downstream functions [121]. However, acquired resistance to EGFR-TKIs in the metastatic setting is unavoidable. While the average progression-free survival (PFS) is between 10 and 16 months, treatment duration can last as short as 1 month [122]. Drug resistance therefore still remains a problem and new therapies and strategies must be developed to overcome such resistance.

*miR-30b/c and miR-221/222*. EGF and MET receptors control gefitinib-induced apoptosis and NSCLC tumorigenesis through the downregulation of specific oncogenic miRNAs, miR-30b/c, and miR-221/222 [123]. Using bioinformatics analysis and luciferase assays,* APAF-1* and* BIM *(previously found to play a role in TKI sensitivity [124, 125]) were determined to be direct targets of miR-221/222 and -30b/c. To investigate the roles these miRNAs play in gefitinib-induced apoptosis, wild-type EGFR expressing NSCLC cells (Calu-1 and A549) and cells with EGFR exon-19 deletions (PC9 and HCC827) were utilized. Upon gefitinib treatment, significant downregulation of miR-30b/c and miR-221/222 with an increased BIM and APAF-1 protein levels were observed only in PC9 and HCC827 sensitive cells. To further determine the contribution of miR-30b/c and miR-221/222-mediated APAF-1 and BIM downregulation to cellular TKI response, Garofalo et al. (2012) overexpressed APAF-1 and BIM in A549 resistant cells, which consequently led to gefitinib-induced PARP cleavage. Furthermore, as miR-30b/c and miR-221/222 are regulated by MET, a strong downregulation was observed of these miRNAs when Calu-1- and A549-MET overexpressing cells were treated with MET inhibitors SU11274. Furthermore an increase in caspase-3/7 activity and decreased cell viability was observed in SU11274-treated Calu-1 cells following exposure to varying gefitinib concentrations. Together, these results suggest that MET inhibition restores gefitinib sensitivity in TKI-resistant Calu-1 through downregulation of miR-30b/c and miR-221/222 [123].

*miR-214*. miR-214 is significantly upregulated in gefitinib resistant lung adenocarcinoma cell line, HCC827/GR, in comparison to parental HCC827 lung adenocarcinoma cells. HCC827/GR was obtained by exposing HCC827 cells to increasing concentrations of gefitinib over six months [126]. Using dual-luciferase reporter assay, Wang et al. (2012) confirmed PTEN as a direct functional target of miR-214. PTEN encodes a 403 amino acid dual-specificity lipid and protein phosphatase which functions as a tumor suppressor in many tumors [127, 128]. Knockdown of miR-214 expression resulted in the upregulation of PTEN protein and inactivation of AKT, which is largely linked to antiapoptotic function [129, 130]. Furthermore, knockdown of miR-214 resensitized HCC827/GR to gefitinib, as demonstrated through MTS assay. miR-214 was thus concluded to potentially serve as a therapeutic target to reverse the acquired resistance of gefitinib in lung adenocarcinoma cells.

*miR-133b*. Expression of miR-133b is significantly downregulated in NSCLC tissues in comparison to nonneoplastic lung tissues [131], and the 3′UTR of* EGFR* was found to be a direct target of this miRNA thus inhibiting its expression. Treatment of EGFR-addicted lung cancer cells, PC-9 and A549 with miR-133b mimic inhibited phosphorylation of EGFR, AKT, and extracellular signal-related kinase (ERK)1/2, thus inhibiting their growth and invasion abilities. However in non-EGFR-addicted NSCLC cells NCI-H1650 and NCI-H1975, no significant changes in the expression of phosphorylated EGFR, AKT, and ERK1/2 were found. Furthermore, miR-133b was able to restore or enhance EGFR-TKI sensitivity in NSCLC cells, especially in EGFR-addicted cells. These findings reveal that transfection of miR-133b in EGFR-addicted NSCLC has the therapeutic potential for overcoming EGFR-TKI resistance [131].

### 4.6. PRIMA-1-Related miRNA

The tumor suppressor* p53* gene regulates cell growth through the activation of the transcription of numerous genes specifically those involved in cell cycle regulation, apoptosis, and genomic stability [132–134] andhas also been implicated in the response to anticancer therapies [133].* p53* has been reported to be frequently mutated in humans cancers with mutations occurring in greater than 50% of lung tumors [135, 136]. Restoration of wild-type p53 function has led to regression of cancers in mice [137, 138], and thus efforts to treat cancers through the reactivation of* p53* with a low-molecular-weight compound such as PRIMA-1 (*p53*-dependent reactivation and induction of massive apoptosis) [139, 140] are widely supported.

*miR-34a*. In a study conducted by Duan et al. (2010), the role of miR-34 family members in regulating PRIMA-1 induced apoptosis was investigated. The authors discovered that PRIMA-1 was able to upregulate miR-34a in* p53* mutant cells. Previous studies have shown evidence that the miR-34 family plays a role in the regulation of cell proliferation and apoptosis [19, 141–147]. The results of this study suggest that PRIMA-1 is able to restore wild-type function to mutant* p53*, which will upregulate miR-34a to induce apoptosis in lung cancer cells [148].

### 4.7. Curcumin Related miRNA

Curcumin is a compound extracted from the rhizomes of* Curcuma longa *L. and studies carried out exhibited its diverse pharmacological effects which include anti-inflammatory, antioxidant, and antitumor activities [149]. Previous studies have also shown that curcumin can induce apoptosis in many types of cancer cells [150, 151], through the inhibition of* NF-*κ*B*,* survivin/BIRC5*, and* BCL-2* [152, 153]. However few studies have been carried out to report the importance of miRNA expression modulation in mediating the biological effects of curcumin.

miRNA-186*. In a study conducted by Zhang et al.(2010), curcumin was shown to have the ability to inhibit cell proliferation and induce apoptosis in A549 cells. The authors performed a cluster analysis on the expression profiles on curcumin-treated and dimethyl sulfoxide (DMSO) control-treated samples and found that miR-186* was shown to be significantly downregulated in response to curcumin treatment, thus suggesting that miR-186* may play an oncogenic role in human lung cancer cells. Inhibition of miR-186* was shown to greatly decrease cell proliferation in A549 cells and increase the induction of apoptosis. Furthermore, caspase-10 was revealed to be a direct target of miR-186 [154]. This study thus provided the first evidence that miR-186* is essential for the anticancer effects of curcumin in A549 cells and that caspase-10 may be an important target of miR-186* in preventing apoptosis.

However, even though curcumin has exhibited antitumor activity, there has been concern regarding the effects of curcumin on multidrug resistant cells [155, 156]. To analyze such effects, A549/DDP, the DDP-resistant derivative of parental A549 cells generated by coculturing parental A549 cells with 6 nm DDP to maintain the drug resistance phenotype, was utilized. In a study conducted by Zhang et al. (2010), a comprehensive miRNA profiling of untreated multidrug-resistant cell line (A549/DDP) was performed and compared against results obtained for A549/DDP cells treated with curcumin. Results showed that miR-186* was downregulated more than 2.5-fold compared to levels in control cells. The antiapoptotic effects of miR-186* in A549/DDP cells were investigated and it was found that transfection of miR-186* mimics led to an inhibition of apoptosis in comparison to that in the control, thus suggesting that miR-186* plays an oncogenic role in this cell line. To confirm the role miR-186* plays in curcumin-induced A549/DDP apoptosis, flow cytometry was used to detect the rate of apoptosis in A549/DDP cells treated with curcumin, control cells, or curcumin combined with miR-186* mimic cells. Results indicated that apoptosis in the combination group was significantly decreased in comparison to cells treated with curcumin [157]. These findings reveal that curcumin is able to induce apoptosis in the multidrug resistant cell line by downregulating miR-186*.

### 4.8. Multidrug Resistance

*miR-200bc/429*. In 2012, Zhu et al. reported that the miR-200bc/429 cluster was downregulated in multidrug-resistant A549/DDP cells, in comparison to parental A549 cell [158]. Recent studies have suggested that aberrant DNA methylation of the promoter region of the miR-200bc/429 cluster may be a critical mechanism leading to dysregulated expression level of the miR-200 family [159, 160]. While the roles of the two sequence clusters of miR-200 family on the epithelial-to-mesenchymal transition of tumor cells are well studied, the role that this miRNA family plays on apoptosis has been minimally studied. Zhu et al. demonstrated using MTT that transfection of miR-200bc/429 cluster mimics into A549/DDP greatly enhanced sensitivity of this cell line to various anticancer drugs including vincristine (VCR), etoposide (VP-16), adriamycin (ADR), and DDP. It was found that miR-200bc/429 cluster was able to modulate multidrug resistance (MDR) in lung cancer cell lines, at least in part by inhibiting the antiapoptotic Bcl-2 and XIAP protein expression, thus affecting the mitochondrial release of cytochrome c. Therapeutic methods that target the miR-200bc/429 clusters thus provides a promising method to enhance treatment effect of NSCLC.

*miR-181b*. In another study by Zhu et al. (2010), miR-181b was also found to be downregulated in multidrug-resistant A549/DDP cells, in comparison to parental A549 cell line [161]. To determine whether miR-181b has a direct role in MDR development, MTT assay was performed revealing that all A549/DDP cells transfected with miR-181b mimic exhibited a significant increase in sensitivity to a number of anticancer drugs including 5-fluorouracil (5-Fu), VCR, DDP, VP-16, and ADR. Bioinformatics analysis predicted antiapoptotic* BCL-2* as a potential target of miR-181, with two conserved target sites in the 3′UT region. Transfection of miR-181a in A549/DDP cells led to a significant decrease in Bcl-2 protein levels, as demonstrated by Western blot. Furthermore, A549/DDP miR-181b transfected cells also led to an increase in apoptosis as detected by flow cytometry. Together these results demonstrate miR-181b's ability to modulate the development of MDR in lung cancer cell lines, at least in part, by modulation of apoptosis through the targeting of the antiapoptotic* BCL-2*.

### 4.9. TRAIL-Related miRNAs

The Apo2L/tumor necrosis factor- (TNF-) *α*-related apoptosis inducing ligand (TRAIL) is a member of the TNF family that is known to induce apoptosis in various cancers [162]. Treatment of transformed cells with TRAIL has been shown to successfully induce apoptosis both* in vitro* and* in vivo *[162, 163]; however a wide range of human cancer cells are resistant to TRAIL-induced apoptosis [164].

*miR-221 and -222*. To identify the mechanisms by which miRNAs may play a role in TRAIL resistance, Garofalo et al. (2008) carried out a genome wide profiling of miRNAs in three different lung cancer cell lines (A459, Calu-1, and NCI-H460) and found that miR-221 and miR-222 were markedly upregulated in TRAIL-resistant cells. In TRAIL sensitive cells NCI-H460, TRAIL was able to induce the activation of the caspase cascade, evaluated by the appearance of cleaved fragments. However, transfection of NCI-H460 cells with pre-miRs-221 and -222 caused a significant reduction of TRAIL-mediated cell death machinery activation. Further experiments deduced that miR-221 and-222 directly targeted *p*27^Kip1^, and inhibition of *p*27^Kip1^ via pre-miR-221 and -222 transfection led to an increase in cell resistance to TRAIL as assessed by Annexin V staining, and PARP and caspase-8 activation. Taken together, the authors' results demonstrate that increased levels of miR-221 and -222 may modulate sensitivity of NSCLC cells to TRAIL with important implications in the design of new therapeutic agents.

*miR-34a and miR-34c*. In another study, miR-34a and miR-34c expression were found to be significantly downregulated in NSCLC cells and lung tumors in comparison to normal lung tissues. Performing a bioinformatics search, Garofalo et al. (2013) determined that* PDGFR-*α** and* PDGFR-*β** were targets of these miRNAs; both of which have been reported to be overexpressed and associated with poor outcome in lung cancer [165]. Through targeting* PDGFR-*α** and* PDGFR-*β**, miR-34a/c were able to decrease invasiveness as well as increase TRAIL-induced apoptosis. TRAIL resistance is common in lung tumors and it has been reported that PDGFR-*α* and PDGFR-*β* regulate the PI3K/Akt and ERK1/2 pathways [166, 167], which play a role in TRAIL-induced apoptosis [168]. Phosphorylation levels of ERKs were found to be decreased following ectopic expression of miR-34a/c; additionally caspase-3/7 assay revealed an increase in TRAIL sensitivity. This study demonstrates that inhibition of* PDGFR-*α** and* PDGFR-*β** by miR-34a/c is able to antagonize tumorigenicity and increase sensitivity to TRAIL-induced cell death [169].

*miR-212*. PED/PEA-15 is a death effector domain (DED) family member, which has been implicated in the processes of cell growth and metabolism [170–172]. Furthermore, PED/PEA-15 has a broad range of antiapoptotic ability, being able to inhibit both the intrinsic and extrinsic apoptotic pathways [171, 173]. Zanca and colleagues (2008) reported that PED/PEA-15 overexpression plays a role in TRAIL resistance in NSCLC [174]; however the mechanism that regulates its expression is not well known. In further studies, Incoronato et al. (2010) reported that NSCLC-affected lung tissue has an increased expression of PED/PEA-15 with a concurrent downregulation of miR-212 and decreased response to TRAIL treatment [175]. miR-212 negatively regulates* PED/PEA-15* by directly binding to its 3′UTR. miR-212 downregulation has previously been reported to be involved in lung cancer response to chemotherapy, in particular to docetaxel [101]. In this study, transfection of NSCLC Calu-1 cells with pre-miR-212 led to a decrease in PED/PEA-15 expression with increased caspase-8 activation following treatment with TRAIL, indicating increased sensitivity of Calu-1 cells to TRAIL-mediated cell death. Therefore, the expression of miR-212 could be used to predict therapeutic response to TRAIL in lung cancer.

## 5. Conclusions

In terms of molecular events occurring in tumors, evasion of apoptosis is an important hallmark of tumor progression. Recent evidence has exhibited deregulated miRNAs to play a role in the apoptotic process. In lung cancer, upregulated miRNAs have been shown to serve as oncogenes, targeting tumor suppressor, and/or proapoptotic genes, while downregulated miRNAs can function as tumor suppressors, targeting oncogenic and/or antiapoptotic genes. Additionally, studies have also indicated that miRNAs play a significant role in altering sensitivity and resistance to cytotoxic treatment. Targeting of specific miRNAs could therefore potentially be used as valuable therapeutics for lung cancer. Together, these studies have illustrated the importance for further studies and validation of miRNAs and their targets. Furthermore, there is a serious shortage in research being carried out in miRNA-regulated apoptosis in SCLC. As SCLC accounts for 16.8% of lung cancer incidence and is a highly aggressive form of lung cancer it would be of great interest to determine the functions of miRNAs in regulation of apoptosis in this lung cancer subtype.

## Figures and Tables

**Table 1:** Upregulated apoptosis-associated miRNAs in lung cancer.

MicroRNA	Target genes	Function	Cell lines	*In vivo* models	Citation
miR-197	BMF, NOXA	Repress *p53*-dependent apoptotic cascade miR inhibition decreases cell viability miR inhibition impairs cell growth and anchorage-independent colony formation	A549, Calu-1, NIH-H460, NCI-H1299	Nude mice	[35]

miR-21	SPRY1, SPRY2, BTG2, PDCD4, APAF1, FasL, RHOB	Enhance tumor proliferation and survival Inhibit apoptosis miR deletion suppresses Ras-driven transformation	N/T	K-ras^LA2^ mice CAG-miR-21 transgenic mice CAG-miR-21; K-ras^LA2^ compound mutant mice	[37]

miR-212	AChE	Prevent apoptosis Maintain cell proliferation capacity Modulate CDDP-induced NSCLC cell apoptosis	HEK-293T, NCI-H520, NCI-H460, SK-MES-1, BEAS-2B	Nude mice	[43]

miR-17-5p, miR-20a	E2F1	miR inhibition reduces of cell growth. miR inhibition induces apoptosis and increases proportions of sub-G1 populations.	Calu-6, A549, ACC-LC-172	N/T	[45]

N/D: not determined; N/T: not tested.

**Table 2:** Downregulated apoptosis-associated miRNAs in lung cancer.

MicroRNA	Target genes	Function	Cell lines	*In vivo* models	Citation
miR-7	BCL-2	Suppress cell proliferation and induce cell apoptosis Inhibit cancer cell migration *in vitro* Reduce tumorigenicity *in vivo *	A549, NCI-H1299, NCI-H1355, NCI-H460, MRC-5, HEK-293T	Nude mice	[51]

miR-198	FGFR1	Inhibit lung cancer cells proliferation Enhance cell apoptosis Inhibits growth of tumor graft in nude mouse	A549, NCI-H460	Athymic BALB/c nude mice.	[69]

miR-451	RAB14	Inhibit *in vitro* proliferation and enhance apoptosis Decrease phosphorylation of AKT and increased BAX or Bad protein level Associated with *in vivo* proliferation capacity	A549, SPC-A1, NCI-H520	Athymic BALB/c nude mice.	[75]

miR-192	RB1	Inhibit cell proliferation and promotes cell apoptosis Arrest cell in G1 phase Inhibit tumorigenesis *in vivo *	A549, NCI-H460, 95D	Athymic BALB/c nude mice.	[76]

miR-335	BCL-W, SP1	Suppress proliferation and invasion ability of cells Induce apoptosis Suppress metastasis and invasiveness of cells	A549, NCI-H1299	N/T	[52]

miR-608	N/D	Increase cell death in *Bcl-xL* silenced cells	A549, SK-LU-1	N/T	[55]

miR-203	PKC*α*	Decrease cell proliferation Promote cell apoptosis, but this effect only partially relies on its downregulation of PKC*α*	A549	N/T	[61]

miR-413	PKC*ε*	Inhibit cell proliferation and enhance apoptosis	A549, Calu-1	N/T	[63]

miR-146a	EGFR	Inhibit cell growth and induces cell apoptosis Suppress motility Enhance cell proliferation inhibitory effect of TKIs and cetuximab	NCI-H358, NCI-H1650, NCI-H1975, NCI-H292, HCC827	N/T	[70]

miR-26a	EZH2	Inhibit cell proliferation *in vitro*Block G1/S phase transition and induced apoptosis Decreased metastasis capacity and invasion	SPC-A1, A549, SK-MES-1	N/T	[71]

N/D: not determined; N/T: not tested.

**Table 3:** Drug-associated miRNAs in lung cancer.

Drug	MicroRNA	Target genes	Function	Cell Lines	*In vivo* models	Citation
Docetaxel	miR-100	Plk1	Chemosensitize lung adenocarcinoma cells to docetaxel Suppress cell proliferation, enhance apoptosis and cell cycle arrest in the G2/M phase of cell cycle Down-regulate *in vivo* cell proliferating ability	SPC-A1, A549, NCI-H1299, SPC-A1/DTX	Nude mice	[102]
	miR-650	ING4	Confer docetaxel chemoresistance both *in vitro* and *in vivo *	SPC-A1, NCI-H1299	Athymic BALB/c nude mice	[105]

Cisplatin	miR-451	N/D	Enhance DDP chemosensitivity Inhibit growth and enhance apoptosis	A549	BALB/c nude mice	[84]
	miR-31	ABCB9	Induced DDP resistance Antiapoptotic effect	SPC-A1, LTEP-A2, NCI-H460, NCI-H1299	N/T	[86]

Paclitaxel	miR-133a/b, miR-361-3p	N/D	Oncogenic miR inhibition reduces cell survival	NCI-H1155, NCI-H1993, NCI-H358	N/T	[91]
	miR-101	EZH2	Decrease proliferation and invasive ability of cells Sensitize cells to paclitaxel	NCI-H226, A549, NCI-H358, 801D	N/T	[98]

Gemcitabine	miR-133b	MCL-1, BCL-W	Increase apoptosis Gemcitabine sensitivity	A549, NCI-H23, NCI-H2172, NCI-H226, NCI-H522, NCI-H2009, NCI-H1703	N/T	[111]

EGFR-TKI	miR-30b/c miR-221/222	APAF-1, BIM	Gefitinib-induced PARP cleavage Decrease cell viability Restore gefitinib sensitivity	Calu-1, A549, PC-9, HCC827	N/T	[123]
	miR-214	PTEN	Oncogenic Knockdown sensitizes cells to gefitinib	HCC827, HCC827/GR	N/T	[126]
	miR-133b	EGFR	Inhibit cell's growth and invasion abilities Enhance EGFR-TKI sensitivity	PC-9, A549, NCI-H1650, NCI-H1975	N/T	[131]

Curcumin	miRNA-186*	Caspase-10	Inhibits cell apoptosis Down-regulation of miR-186 by curcumin induces apoptosis	A549, A549/DDP	N/T	[154, 157]

TRAIL	miR-221, miR-222	N/D	Impair TRAIL-dependent apoptosis Induce TRAIL-resistance	Calu-1, A549, NCI-H460	N/T	[168]
	miR-34a, miR-34c	PDGFR-*α*, PDGFR-*β*	Augment TRAIL response Reduce migratory and invasive capacity of cells	NCI-H460, A549, NCI-H1299, Calu-6 NCI-H1703,	N/T	[169]
	miR-212	PED	Increase sensitivity to TRAIL Tumor suppressor	Calu-1, NCI-H460	N/T	[175]

PRIMA-1	miR-34a	N/D	Induce apoptosis in the lung cancer cells containing mutant *p53 *	A549, NCI-H211, NCI-H1155, NCI-H1299	N/T	[148]

Multidrug Resistance	miR-200bc/429	BCL2, XIAP	Enhanced sensitivity to various anticancer drugs including VCR, CDDP, VP-16, and ADR	A549, A549/CDDP	N/T	[158]
	miR-181b	BCL2	Increased sensitivity to a number of anticancer drugs including VCR, 5-Fu, CDDP, VP-16, and ADR	A549, A549/CDDP	N/T	[161]

N/D: Not determined, N/T: Not tested.

## Abbreviations

*γ*-H2AX: Gamma, H2A histone family, member X
3′UTR: 3′-Untranslated region
5-Fu: 5-Fluorouracil
ABCB9: ATP-binding cassette, subfamily B (MDR/TAP), member 9
AChE: Acetylcholinesterase
ADR: Adriamycin
Akt: Protein kinase B
Apaf-1: Apoptotic peptidase activating factor 1
BAK: BCL2-antagonist/killer 1
BAX: BCL2-associated X protein
BCL-2: B-cell CLL/Lymphoma
BCL-W: BCL2-like 2
BCL-XL: BCL2-like 1
BIM: BCL2-like 11 (apoptosis facilitator)
BIRC5: Baculoviral IAP repeat containing 5
BMF: Bcl-2-modifying factor
BTG2: BTG family, member 2
DAB2IP: Disabled homolog 2-interacting protein
DDP: Cisplatin
DMSO: Dimethyl sulfoxide
E2F1: E2F transcription factor 1
EGFR: Epidermal growth factor receptor
ERK1: Mitogen-activated protein kinase 3
ERK2: Mitogen-activated protein kinase 1
EZH2: Histone-lysine N-methyltransferase
FasL: Fas ligand
FDA: Food and drug administration
FGFR1: Fibroblast growth factor receptor 1
FITC: Fluorescein isothiocyanate
HTS: High-throughput screening
I*κ*B*α*: Nuclear factor of kappa light polypeptide gene enhancer in B cells inhibitor alpha
ING4: Inhibitor of growth family, member 4
IRAK-1: Interleukin-1 receptor-associated kinase 1
MAPK: Mitogen activated protein kinase
MCL1: Molecule myeloid leukemia 1
MDR: Multidrug resistance
miRNA: MicroRNA
NF-*κ*B: Nuclear factor kappa-light-chain-enhancer of activated B cells
NSCLC: Non-small cell lung cancer
NOXA: Phorbol-12-myristate-13-acetate-induced protein 1
*p*27^Kip1^: Cyclin-dependent kinase inhibitor 1B
p53: Tumor protein p53
PDCD4: Programmed cell death 4 (neoplastic transformation inhibitor)
PDGFR-*α*: Platelet-derived growth factor receptor-alpha polypeptide
PDGFR-*β*: Platelet-derived growth factor receptor-beta polypeptide
PEA-15: Astrocytic phosphoprotein PEA-15
PED: Preimplantation embryonic development
PI3K/AKT: Phosphatidylinositol 3-kinase/protein kinase B
PKC: Protein kinase C
PKC-*α*: Protein kinase C-alpha
PKC-*ε*: Protein kinase C-epsilon
PLK1: Polo-like kinase 1
PRIMA-1: * p53*-dependent reactivation and induction of massive apoptosis
PFS: progression-free survival
PUMA: BCL2 binding component 3
RAB14: RAB14, member RAS oncogene family
RB1: Retinoblastoma 1
RHOB: Ras homolog family member B
RUNX3: Runt-related transcription factor 3
SCLC: Small cell lung cancer
SP1: Specificity Protein 1
SPRY1: Sprouty homolog 1, antagonist of FGF signaling (*Drosophila*)
SPRY2: Sprouty homolog 2 (*Drosophila*)
STAT: Signal transducer and activator of transcription
TRAIL: Apo2L/tumor necrosis factor- (TNF-) *α*-related apoptosis inducing ligand
TGF-*β*: Transforming growth factor-beta
VCR: Vincristine
VP-16: Etoposide
WNT: Wingless-type MMTV integration site family
XIAP: X-linked inhibitor of apoptosis.
